# Editorial: Influence of diet and maternal environment on neuropsychological development early in life

**DOI:** 10.3389/fpubh.2024.1401154

**Published:** 2024-06-03

**Authors:** Roberta Zupo, Ferdinando Murgia, Rodolfo Sardone, Luigi Vimercati, Emilia Matera, Maria Lisa Clodoveo

**Affiliations:** ^1^Department of Interdisciplinary Medicine (DIM), University of Bari Aldo Moro, Bari, Italy; ^2^Department of Obstetrics and Gynecology, “Miulli” General Hospital, Bari, Italy; ^3^Unit of Statistics and Epidemiology, Local Health Authority of Taranto, Taranto, Italy; ^4^Department of Eye and Vision Sciences, University of Liverpool, Liverpool, United Kingdom; ^5^Section of Occupational Medicine, Interdisciplinary Department of Medicine, University of Bari, Bari, Italy; ^6^Department of Precision and Regenerative Medicine and Ionian Area (DiMePRe-J), University of Bari Aldo Moro, Bari, Italy

**Keywords:** maternal environment, public health, diet, neuropsychological development, nutrition

The prenatal and perinatal environment, along with the eating habits of early life, may shape the neuropsychological developmental trajectories of infants and future adolescents (1). Epidemiology shows that the number of people worldwide featuring neurodevelopmental disorders (NDDs), which include ADHD, autism, learning disabilities, intellectual disabilities, and behavioral disorders, has increased over the past decade (2). Taking action on modifiable factors could have far-reaching effects on public health practice and policy development. Data from a recent meta-analysis (3) found a median prevalence per 1,000 people for any NDD of 7.6 (95%CI 7.5–7.7), 11.3 (11.7–12.0) for neurological disorders, and 3.2 (95%CI 3.1–3.3) for mental conditions such as ADHD.

In this context, this Research Topic aimed to add knowledge to the understanding of the composite network of direct and indirect environmental effects on the neuropsychological health of children and adolescents. The baseline assumption is that different and complex environmental dimensions, including maternal environment, physical and chemical environment (e.g., from exposure to specific pollutants to exposure to multiple and possibly interacting substances) (4–6), socioeconomic environment (e.g., education, social support, stress, work environment, and access to health care), and living environment (e.g., maternal and fetal diet, housing conditions, green space, and exposure to noise and light) (1, 7) are among the modifiable factors within the exposome still under study.

Four articles, including one meta-analysis, were included in this Research Topic. Two original research papers focused on the effect of maternal supplementation in the perinatal period, analyzing the effect of folic acid on autism spectrum disorders (Jiang et al.) and dietary fish consumption (Inoue et al.) on neurodevelopmental features at age 3. One study carried out a meta-analysis of RCTs that analyzed the effect of probiotic supplementation in children and adolescents on neurocognitive outcomes (Lin et al.). Finally, a study conducted in Bangladesh analyzed the effect of environmental temperature on abortion risk (Das et al.).

Using data from the Japan Environment and Children's Study (JECS), a national survey, Inoue et al. conducted a quintile logistic regression analysis to assess the association between maternal fish intake during pregnancy and child neurodevelopment at age 3 years in 91,909 mother-child pairs. Results showed that maternal fish intake during pregnancy was independently associated with a reduced risk of delay in problem-solving skills at 6 months of age and in motor and problem-solving skills at 1 year of age. The researchers concluded that fish consumption during pregnancy may be associated with a reduced risk of neurodevelopmental delay in 3-year-old children, particularly in the fine motor, problem-solving, and personal-social domains. However, further longitudinal investigations after age 3 could further clarify the association.

Jiang et al. studied the cross-sectional relationship between maternal folic acid supplementation during the pre-conceptional and prenatal periods and the subsequent risk of autism spectrum disorder in offspring. A total of 6,049 children aged 16–30 months were recruited (2016–2017) in China. Parents of enrolled children provided information on maternal supplemental folic acid intake, socio-demographic information, and related covariates. Findings showed that compared with mothers who consistently used folic acid supplements from pre-conception to the prenatal period, those who never used folic acid supplements were statistically significantly associated with a nearly 3-fold higher risk of autism spectrum disorder in their offspring (OR = 2.88).

Lin et al. conducted a meta-analysis of randomized controlled trials (RCTs) investigating the therapeutic efficacy of probiotics in improving cognitive function to provide up-to-date evidence of the therapeutic effects of probiotics on improving various neurocognitive functions in infants and children, as well as to identify important factors that may influence the efficacy of their treatment. Nine RCTs involving a total of 3,026 participants were brought into review. Analyses of studies that offered a course of probiotic treatment of more than 6 months demonstrated a significantly better neurocognitive outcome than placebos, although this result was based on only two RCTs involving a total of 451 participants.

Das et al. conducted a study in a coastal region of Bangladesh (2012–2020) documenting 23,482 pregnancies among 13,376 women followed for their pregnancy outcomes. Temperature records were obtained from the Bangladesh Meteorological Department's weather station in Cox's Bazar. Pregnancy outcome dates were related to the daily mean temperature on the day of pregnancy outcome. A logistic regression model was used to examine the relationship between temperature and incidence of miscarriage. Results showed that miscarriages peaked between 8 and 14 weeks of gestation and varied with temperature. For women exposed to temperatures between 28°C and 32°C, the risk of miscarriage was 25 percent higher than for those exposed to temperatures from 16°C to 21°C. Ultimately, the study establishes a connection between miscarriage and high environmental temperatures in a coastal region of Bangladesh. From a preventive perspective, the implementation of timely and appropriate adaptation strategies to prevent miscarriage is of paramount importance, especially in countries with high population density.

To sum up, when it comes to modifiable factors in the context of neurodevelopmental disorders, pre- and perinatal nutrition is critical. Much room still lies for longitudinal evidence fit to explain biological mechanisms underlying the connections between exposome and trajectories of neurodevelopmental outcomes in early life.

## Author contributions

RZ: Writing – original draft. FM: Writing – original draft. RS: Writing – review & editing. LV: Writing – review & editing. EM: Writing – review & editing. MC: Writing – review & editing.
